# Perceptions and experiences of living with dementia in sub‐Saharan Africa: a systematic review of qualitative studies

**DOI:** 10.1002/alz.093077

**Published:** 2025-01-09

**Authors:** Maëlenn Guerchet, Alexandre Boyer, Pierre‐Marie Preux

**Affiliations:** ^1^ Inserm U1094, IRD UMR270, Univ. Limoges, CHU Limoges, EpiMaCT ‐ Epidemiology of chronic diseases in tropical zone, Institute of Epidemiology and Tropical Neurology, OmegaHealth, Limoges France; ^2^ LEMACEN, Faculty of Health Sciences, University of Abomey‐Calavi, Cotonou, Other Benin; ^3^ Inserm U1094, IRD UMR270, Univ. Limoges, CHU Limoges, EpiMaCT, Limoges, NA France; ^4^ Inserm U1094, IRD UMR270, Univ. Limoges, CHU Limoges, EpiMaCT, Limoges France

## Abstract

**Background:**

In sub‐Saharan Africa (SSA), the number of people living with dementia is expected to double every 20 years, from 2.7 to 7.6 million. Understanding beliefs, perceptions and experiences relating to dementia, care, and help‐seeking are critical to understanding the nature of the problem and designing research programmes, interventions and services which meet locally defined needs. We carried out a systematic review of the scientific qualitative evidence on the perceptions and experience of living with dementia in SSA.

**Method:**

We conducted a systematic review of qualitative studies (open‐ended in‐depth interviews, focus‐group discussions and ethnographic methods) including people living with dementia (PLWD), family caregivers of PLWD, paid caregivers, healthcare workers, traditional and faith healers with experience of treating PLWD, and community members. The search strategy to identify relevant articles used the search terms “(dementia OR Alzheimers)” AND “(qualitative OR anthrop* OR ethnog* OR cultural OR understand* OR belie* OR experien* OR phenomenol*) AND “(Africa OR sub‐Saharan Africa OR [list of countries included in SSA according to World Bank definition]), in eight databases (PubMed, BiblioInserm, PsycINFO, PsycARTICLE, Web of science, Google Scholar, IENT et DATAD – Database of African Theses and Dissertation). A thematic synthesis approach was then used.

**Result:**

The search retrieved 4967 abstracts. Following abstract screening and full‐text assessment, 16 studies were eligible from 10 different countries (South Africa, Tanzania, Nigeria, Ghana, Republic of Congo, Central African Republic, Kenya, Ethiopia, Uganda, and Burkina Faso). Overall, there was a lack of awareness of dementia among the community and no equivalent term for dementia was identified in any local languages. Often it was believed that although not experienced by everyone, dementia‐like symptoms were part of “normal ageing”. Participants frequently reported problems with memory and communication as well as problems typically associated with co‐morbidities of old age (pain, sight/hearing problems).

**Conclusion:**

Qualitative evidence regarding dementia perceptions and experiences in African populations remains limited compared to quantitative evidence. Findings suggest that there is a desire to gain a better understanding of the lived experience of dementia, which is essential to design culturally sensitive education, to counteract stigma and to enhance management of the condition.

